# Viral Linkage in HIV-1 Seroconverters and Their Partners in an HIV-1 Prevention Clinical Trial

**DOI:** 10.1371/journal.pone.0016986

**Published:** 2011-03-02

**Authors:** Mary S. Campbell, James I. Mullins, James P. Hughes, Connie Celum, Kim G. Wong, Dana N. Raugi, Stefanie Sorensen, Julia N. Stoddard, Hong Zhao, Wenjie Deng, Erin Kahle, Dana Panteleeff, Jared M. Baeten, Francine E. McCutchan, Jan Albert, Thomas Leitner, Anna Wald, Lawrence Corey, Jairam R. Lingappa, for the Partners in Prevention HSV/HIV Transmission Study Team

**Affiliations:** 1 Department of Medicine, University of Washington School of Medicine, Seattle, Washington, United States of America; 2 Department of Microbiology, University of Washington School of Medicine, Seattle, Washington, United States of America; 3 Department of Laboratory Medicine, University of Washington School of Medicine, Seattle, Washington, United States of America; 4 Department of Biostatistics, University of Washington School of Medicine, Seattle, Washington, United States of America; 5 Department of Global Health, University of Washington School of Medicine, Seattle, Washington, United States of America; 6 Bill & Melinda Gates Foundation, Seattle, Washington, United States of America; 7 Department of Microbiology, Tumor and Cell Biology, Karolinska Institute, Stockholm, Sweden; 8 Los Alamos National Laboratory, Los Alamos, New Mexico, United States of America; 9 Department of Epidemiology, University of Washington School of Medicine, Seattle, Washington, United States of America; 10 Department of Pediatrics, University of Washington School of Medicine, Seattle, Washington, United States of America; 11 Vaccine and Infectious Disease Division, Fred Hutchinson Cancer Institute, Seattle Washington, United States of America; The University of Hong Kong, Hong Kong

## Abstract

**Background:**

Characterization of viruses in HIV-1 transmission pairs will help identify biological determinants of infectiousness and evaluate candidate interventions to reduce transmission. Although HIV-1 sequencing is frequently used to substantiate linkage between newly HIV-1 infected individuals and their sexual partners in epidemiologic and forensic studies, viral sequencing is seldom applied in HIV-1 prevention trials. The Partners in Prevention HSV/HIV Transmission Study (ClinicalTrials.gov #NCT00194519) was a prospective randomized placebo-controlled trial that enrolled serodiscordant heterosexual couples to determine the efficacy of genital herpes suppression in reducing HIV-1 transmission; as part of the study analysis, HIV-1 sequences were examined for genetic linkage between seroconverters and their enrolled partners.

**Methodology/Principal Findings:**

We obtained partial consensus HIV-1 *env* and *gag* sequences from blood plasma for 151 transmission pairs and performed deep sequencing of *env* in some cases. We analyzed sequences with phylogenetic techniques and developed a Bayesian algorithm to evaluate the probability of linkage. For linkage, we required monophyletic clustering between enrolled partners' sequences and a Bayesian posterior probability of ≥50%. Adjudicators classified each seroconversion, finding 108 (71.5%) linked, 40 (26.5%) unlinked, and 3 (2.0%) indeterminate transmissions, with linkage determined by consensus *env* sequencing in 91 (84%). Male seroconverters had a higher frequency of unlinked transmissions than female seroconverters. The likelihood of transmission from the enrolled partner was related to time on study, with increasing numbers of unlinked transmissions occurring after longer observation periods. Finally, baseline viral load was found to be significantly higher among linked transmitters.

**Conclusions/Significance:**

In this first use of HIV-1 sequencing to establish endpoints in a large clinical trial, more than one-fourth of transmissions were unlinked to the enrolled partner, illustrating the relevance of these methods in the design of future HIV-1 prevention trials in serodiscordant couples. A hierarchy of sequencing techniques, analysis methods, and expert adjudication contributed to the linkage determination process.

## Introduction

Characteristics of the transmitting and seroconverting partner affect HIV-1 sexual transmission risk, making studies of HIV-1 serodiscordant couples (in which one partner is HIV-1 infected and the other is uninfected) of significant scientific value [1]. However, the virus is not bound by vows of fidelity and behavioral characteristics of sexual partnerships are often difficult to ascertain due to recall bias and willingness of some persons to report sensitive behaviors such as concurrent partnerships. HIV-1's high mutational capacity has allowed the use of sequencing and phylogenetic analysis, particularly of the envelope gene [2], [3], to objectively identify HIV-1 source partners and to investigate patterns of HIV-1 transmission.

Observational studies have used molecular approaches to study the transmission of HIV-1 from person to person [4], [5] and within populations [6], [7]. Forensic investigations of HIV-1 transmission have also relied upon phylogenetic analysis of HIV-1 sequence data from suspects and victims [8]–[11] (reviewed in [12], [13]) and have required the highest burden of proof (‘beyond a reasonable doubt’) to establish HIV-1 transmission linkage. In contrast, viral sequence-based linkage determination rarely is applied to HIV-1 prevention trials, since most prevention strategies focus on HIV-uninfected persons (e.g., vaccines, microbicides or pre-exposure prophylaxis) in which it is assumed that the efficacy of the intervention is independent of the source of the transmitted virus. However, evaluation of interventions aiming to reduce HIV transmission from infected individuals to their partners require that each seroconversion event be linked to the source partner, as the efficacy of such interventions can only be measured if HIV-1 transmission events are definitively linked to a source partner receiving either the intervention or placebo. Furthermore, definitive linkage information is important for epidemiologic studies seeking to quantify the proportion of HIV-1 infections that could be averted through interventions targeting stable couples or to characterize risk factors associated with those transmissions.

An example of such a prevention trial is the Partners in Prevention HSV-2/HIV-1 Transmission Study that enrolled African HIV-1 serodiscordant heterosexual couples to evaluate the efficacy of herpes simplex virus type 2 (HSV-2) suppression with acyclovir given to HIV-1/HSV-2 dually-infected participants in reducing HIV-1 transmission to their HIV-1-uninfected heterosexual partners [14]. Despite the high probability that HIV-1 strains from couples enrolled in this study would be linked, the clinical trial demanded that HIV-1 transmission endpoints be defined systematically. In this paper we report on our evaluation of genetic linkage between HIV-1 sequences from epidemiologically linked partnerships in the trial and discuss its potential value for future studies.

## Methods

### The Partners in Prevention HSV/HIV Transmission Study

The study design, recruitment, baseline characteristics and primary study findings of the Partners in Prevention HSV/HIV Transmission Study are detailed elsewhere [14]–[16]. Briefly, 3,408 HIV-1 serodiscordant heterosexual couples were enrolled at 14 sites in 7 sub-Saharan African countries. Written, informed consent was obtained from all participants and the research was conducted according to the principles in the Declaration of Helsinki. The University of Washington Human Subjects Review Committee and ethical review committees at each local and collaborating organization approved the Partners in Prevention HSV/HIV Transmission Study protocol and the trial was registered in ClinicalTrials.gov (#00194519). HIV-1 infected partners, all of whom were also infected with HSV-2 and 68% of whom were female, were randomized to either acyclovir (400 mg orally twice-daily) or placebo and followed for up to 24 months. 155 HIV-1 seroconversions, of which 60% occurred in couples with females as HIV-1 infected partners, were detected using rapid and enzyme-linked serologic assays at the local research sites [15], 151 of which were confirmed by HIV-1 Western blot and quantitative HIV-1 RNA measurements at the University of Washington [14]. Of these, nineteen seroconverting partners had negative HIV-1 Western blot but detectable HIV-1 RNA at the time of enrollment (identified by an ‘SC’ in the partner-pair identifier); these were not included as clinical trial endpoints due to HIV-1 acquisition at the time of randomization, but they are included in this report of the linkage analysis. The primary trial endpoint was defined as incident HIV-1 infection in a previously HIV-1 uninfected partner (‘seroconverting partner’) confirmed to be genetically linked to his/her putative transmitting partner (‘HIV-1 infected partner’) by viral sequence analysis.

### Overview of transmission linkage methods

For each putative HIV-1 transmission pair, consensus sequencing of a population of partial HIV-1 *env* and *gag* genes was performed on blood plasma collected within 3 months of seroconversion from both partners. This protocol of sequencing both *env* and *gag* was followed with the exception of 12 pairs that met linkage criteria by *env* sequencing alone; *gag* was not sequenced for these pairs due to time and budgetary constraints. In light of data showing viral genetic homogeneity in most individuals with acute/early HIV-1 infection [17]–[23] it was decided that deeper sequencing on the HIV-1 infected partner would be performed to identify potentially low-level transmitted variants in cases of initially unlinked or indeterminate pairs eligible for inclusion as trial endpoints. For most pairs (42/49) whose sequences did not show clear evidence of linkage by *env* or *gag* consensus sequencing, multiple single molecule (SM) C2-V3-C3 *env* sequences were obtained following endpoint dilution of cDNA from the HIV-1 infected partner's plasma to identify linked variants present at lower frequency. Furthermore, for a subset of unlinked pairs, we performed *env* amplicon pyrosequencing of the HIV-1 infected partner's virus population to detect even rarer variants that may have been transmitted. To provide phylogenetic context for the partners' HIV-1 sequences we collected publicly available subtype A, C, and D sequences from African countries, using 1 sequence per individual, from the Los Alamos National Laboratory HIV Sequence Database (HIVDB) (http://www.hiv.lanl.gov/content/sequence/HIV/mainpage.html). To better characterize the HIV strains circulating in the community at sites with fewer than 10 study-related seroconversions, we obtained *env* and *gag* from 3–8 additional HIV-1 infected individuals enrolled at such sites (N = 32 across all study sites, shown in Table 1 and Supplementary Table S1) who were epidemiologically unlinked to the putative transmission pairs, as a “local control” (LC) comparator population. This was achieved at all but 1 study site (Site 4). An adjudication committee of 3 experts, blinded to randomization arm, independently reviewed sequence data to assign linkage classification as described below. Supplementary Figure S1 shows an overview of these laboratory and analysis methods.

**Table 1:** Transmission pairs and local control participants evaluated and linkage findings by study site and region.

Region	Country	Site	# Putative Transmission Pairs	# Pairs Unable to be Evaluated	# Pairs with Monophyly in *env* or *gag* & Post. Prob. ≥50%	*env* Subtype (# participants by subtype)*				*gag* Subtype (# participants by subtype)*				# Pairs Linked	# Pairs Unlinked	# Pairs Indet.	# Add’l Local Control Participants
						A	C	D	X	A	C	D	X				
East Africa (EA)	Kenya	1	6	0	6	6	0	4	0	7	0	2	3	6	0	0	5
		2	42	2	26	59	9	8	3	56	3	7	13	26	14	0	0
		3	11	0	8	17	1	4	0	15	0	2	4	8	2	1	0
		4	5	0	5	10	0	0	0	10	0	0	0	5	0	0	0
	Tanzania	5	2	0	2	4	0	0	0	2	0	0	2	2	0	0	8
	Uganda	6	30	2	21	28	5	22	2	31	0	16	6	21	7	0	0
EA Subtotal			96	4	68	124	15	38	5	121	3	27	28	68	23	1	13
Southern Africa (SA)	Botswana	7	7	0	4	0	13	0	0	0	14	0	0	4	3	0	3
	South Africa	8	15	0	10	0	29	0	0	0	30	0	0	9	5	1	0
		9	6	0	3	0	12	0	0	0	6	0	0	5	1	0	4
		10	5	0	5	0	10	0	0	0	8	0	0	3	2	0	5
	Zambia	11	10	0	6	3	17	0	0	0	13	0	0	6	3	1	0
		12	3	0	2	0	6	0	0	0	4	0	0	2	1	0	7
		13	13	0	11	0	26	0	0	0	22	0	0	11	2	0	0
SA Subtotal			59	0	41	3	113	0	0	0	97	0	0	40	17	2	19
Total			155	4	109	127	128	38	5	121	100	27	28	108	40	3	32

(X = other subtype).

The number of participants by subtype may not sum to total participants evaluated due to cases with sequences of >1 subtype or inability to obtain a sequence for *env* or *gag*.

### Laboratory methods for HIV-1 sequencing

Technicians were blinded to specimen identification and partnerships. To minimize the risk of specimen mix-up and contamination, laboratory work on HIV-1 infected and seroconverting participants was physically and temporally separated. All pre-PCR steps were performed in PCR clean rooms. Viral RNA was extracted from blood plasma using the Qiagen RNA Blood Mini Kit (Qiagen, Valencia, CA). cDNA was synthesized with Superscript III reverse transcriptase (Invitrogen, Carlsbad, CA) and primers RT2 (HXB2 coordinates 3301–3321) and Nef3 (9015–9038). This was followed by nested PCR targeting *gag* (p17–p24 region) and envelope (*env*, C2-V3-C3 region). We used Expand High Fidelity polymerase (Roche Applied Science, Indianapolis, IN) and primers gag1 (772–793), RT2 (3301–3321), ED3 (5957–5986), and Nef3 (9015–9038) for the multiplexed first round and Taq polymerase (Invitrogen) and primers gag2 (793–818), gag5 (1826–1847), ED31 (6817–6845), and ED33 (7360–7381) for the independent second round reactions. Final sequence lengths were thus ∼1,009 base pairs (bp) for *gag* and ∼516 bp for *env*. For SM sequencing on HIV-1 infected partners' plasma, we used endpoint serially diluted cDNA to ensure that PCR amplification of the targeted regions would originate from a single amplifiable template [24], [25]. During the first part of the study, clonal sequencing of the C2-V5 region of *env* was performed for all pairs (PP1-58), using ED31 (6817–6845) and BH2 (7697–7725) for the first round and DR7 (6990–7021) and DR8 (7638–7668) for the second round. These amplicons were cloned into TOPO TA vector (Invitrogen). Given the time and costs of clonal sequencing for each partner in those first 58 transmission pairs, the trial endpoint committee decided to utilize consensus sequencing of partial *env* and *gag* regions to increase efficiency in determining transmission linkage. Both clonal and consensus sequencing was performed on most of these specimens. For efficiency, this redundancy on linked pairs was eliminated later in the study. Plasmid DNA and PCR products were purified with the FastPlasmid Mini kit (Eppendorf, Westbury, NY) and the QIAQuick PCR or gel extraction kit (Qiagen), respectively. Standard dideoxy terminator sequencing was performed at a local facility. Sequence chromatograms were manually edited with Sequencher 4.5 (Gene Codes, Ann Arbor, MI). Amplicon pyrosequencing of C2-V3-C3 *env* (two, ∼220 bp reads from the 5′ and 3′ ends of the ED31/ED33 *env* amplicon were obtained and analyzed separately) was performed on HIV-1 infected partners' plasma using the Roche 454 Genome Sequencer FLX (Roche Diagnostics, Branford, CT). The number of templates sequenced ranged from 60–2000 per specimen, estimated by endpoint dilution PCR [24].

### Phylogenetic and genetic distance analysis

We screened study sequences against our local laboratory database and the HIVDB using ViroBLAST [26] (http://indra.mullins.microbiol.washington.edu/blast/viroblast.php) to identify specimen mixup or laboratory contamination. Viral subtypes were determined using REGA 2.0 (http://dbpartners.stanford.edu/RegaSubtyping/) or the NCBI subtyping tool (http://www.ncbi.nlm.nih.gov/projects/genotyping/formpage.cgi). In December 2007, we collected all high quality *env* and *gag* sequences from the HIVDB of subtypes A, C, and D from the corresponding gene regions we sequenced, 1 per subject, and created separate alignments for subtypes A, C, and D for *env* (N = 172, 250, and 97, respectively by subtype) and *gag* (N = 142, 304, and 90, respectively) using CLUSTALW [27] or MUSCLE [28], followed by manual adjustment to optimize codon alignments in Seaview v3 [29] or MacClade 4.08 [30]. We added each study sequence to the appropriate alignment, in some cases along with the 5 most closely related sequences found in the HIVDB. Maximum likelihood phylogenetic trees and pairwise distances were determined with the DIVEIN web server [31] (http://indra.mullins.microbiol.washington.edu/cgi-bin/DIVEIN) using a generalized time reversible (GTR) model of evolution.

For cases in which pyrosequencing was performed, reads were initially aligned to an HXB2 reference sequence using Mosaik [32]. We removed reads containing ambiguous bases and of read lengths <100 nucleotides, separated those derived from + and – strands, and manually trimmed trailing ends to remove poor quality data. Local realignments were performed using MUSCLE [28] implemented within the Seaview v3 alignment program [29], followed by further manual refinement in Seaview. Perl scripts were written for Mosaik alignment, conversion of. ace files to. fasta alignments, removal of short reads and those containing N's, sorting alignments at their 5′ and 3′ ends, and determining pairwise distances to the HIV-1 infected and seroconverter consensus sequences (scripts available upon request).

### Reference datasets

We created two reference sequence datasets from individuals with known linkage status to establish the distributions of linked and unlinked *env* and *gag* sequence pairs. The “linked” dataset was derived from sequences from acutely infected individuals and known transmission pairs. From June 2007- April 2009 when we conducted our data analysis, the publicly available sequence data for heterosexual transmission pairs was limited. To augment the number of sequences in our reference data set, we included data from individuals with a variety of HIV risk factors, including the Multicenter AIDS Cohort Study (MACS) [33], [34] (acute infections) as well as from heterosexual [4], [35], male-to-male (unpublished data and [2]), and mother-to-infant [36], [37] transmission cases. Newly available sequences from adjudicator-confirmed linked partner-pairs from this clinical trial were added to the dataset following each of the interim adjudications. In total, sequences from 35/0, 90/57, 117/104, and 147/148 pairs were obtained in *env* and *gag* for the first through final adjudications, respectively.

The “unlinked” reference dataset was composed of epidemiologically unlinked sequences using a dataset composed of sequences from individuals with no known epidemiologic linkage, including sequences from the HIVDB and from previously adjudicated “unlinked” pairs from this study cohort. This included, in the final analysis, 362/309, 485/474, 186/133 sequences in *gag* and *env* from subtype A, C and D, respectively.

### Bayesian analysis of genetic distances

We developed a Bayesian algorithm to derive an estimate of the probability of linkage between sequences in our cohort based on the reference datasets of pairwise genetic distances for epidemiologically and phylogenetically linked and unlinked individuals described above. The purpose of the Bayesian analysis was to have an objective statistical measure of linkage for each HIV-1 transmission event that could account for the prior probability that HIV-1 sequences from these partner pairs in long-term sexual partnerships would be linked. According to Bayes' theorem, the posterior probability that two sequences are linked (*i.e*., the probability of linkage, given existing data) is a function of the prior probability that they are linked and the distributions of genetic distances from known linked and unlinked sequences described above, as shown in the following equation:
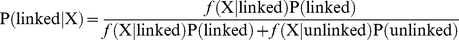



X denotes the objective data obtained during sequence analysis, the pairwise genetic distance in this case. P(linked) and P(unlinked) are the prior probabilities of linkage and lack of linkage for pairs of sequences from HIV-1 infected partner participants in our dataset and f(X | linked) and f(X | not linked) are conditional densities of the genetic distances for linked or unlinked sequences based on the distribution of genetic distances in the reference datasets. As opposed to a ‘pure Bayes’ approach in which an acceptable value or range of values for P(linked) and P(unlinked) are specified, this approach uses an ‘empirical Bayes’ approach. Here, an initial value of P(linked) is chosen (P(linked)  = 0.5), the posterior probabilities of linkage for each couple is computed, and P(linked) is updated as the proportion of partners who are classified as linked in the Partners in Prevention HSV/HIV Transmission Study. This procedure is then iterated until convergence. Prior to adjudication, Bayesian posterior probabilities were calculated using the distance between the 2 most closely related sequences in *env* and *gag* for each partner pair.

### Criteria for assignment of linkage

For each enrolled pair and each level of sequence analysis (consensus, SM and pyrosequencing), HIV-1 linkage was assigned by first requiring that partner-pair derived HIV-1 *env* and/or *gag* sequences form monophyletic clusters (*i.e*., originating from the same terminal node) in maximum-likelihood phylogenetic trees that included sequences from unrelated individuals (“local controls”). Second, the pairwise genetic distances were required to be associated with a Bayesian posterior probability ≥50% for the gene in which monophyly occurred. Partner-pair sequences that met these two requirements were tentatively classified as linked.

An adjudication committee consisting of three independent experts in HIV-1 viral genetics (J.I.M., F.E.M., and initially T.L. and subsequently J.A.), who had not participated in the clinical trial protocol design and were blinded to participants' treatment assignments, evaluated phylogenetic and Bayesian posterior probability for each seroconverter pair. If at least two adjudicators concordantly assigned linkage status, the pair was tentatively classified by that assignment. Pairs with linkage status that could not be determined definitively received indeterminate classifications. Interim adjudication occurred before each meeting of the Partners in Prevention HSV/HIV Transmission Study Data and Safety Monitoring Board and a comprehensive review of the dataset to finalize linkage assignments by consensus was performed before the final clinical trial analysis.

### Statistical analysis

Selected epidemiologic and biological variables were compared in linked and unlinked pairs, evaluating for statistical significance with the two-sided Fisher's exact test for categorical variables and Wilcoxon rank sum test for continuous variables.

## Results

**Figure 1:**
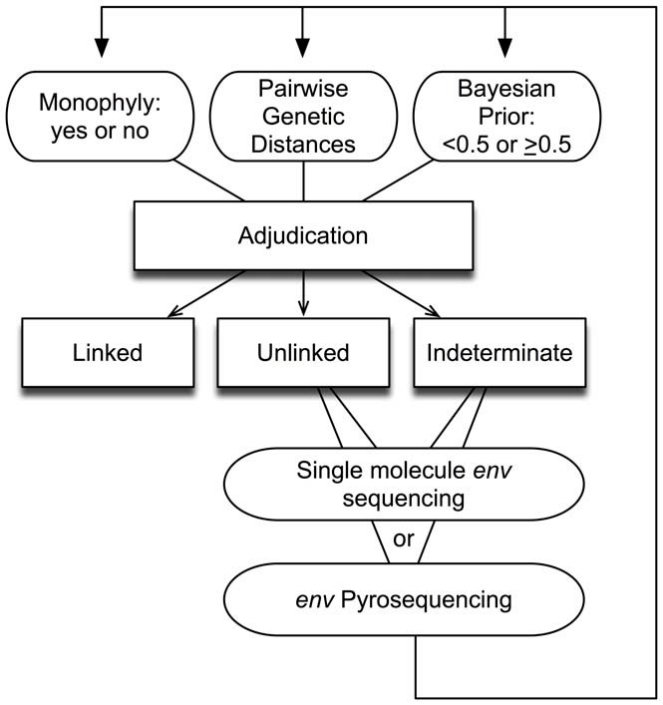
Adjudication criteria used in assigning transmission linkages. For each pair, adjudicators evaluated monophyly (yes/no), genetic distance, and Bayesian posterior probability (≥0.5 or <0.5) and classified the pair as ‘linked’, ‘unlinked’, or ‘indeterminate’. Further evaluation of ‘unlinked’ or ‘indeterminate’ pairs involved gathering additional data, including sequencing of consensus *gag* and/or clonal, single molecule or pyrosequencing of *env*, as well as obtaining sequences from non-transmitting HIV-1 infected participants from the same study site. New trees, distance distributions and Bayesian priors were generated and each pair was re-adjudicated to make final linkage assignments.

During The Partners in Prevention HSV/HIV Transmission Study, 155 incident HIV-1 infections were identified by HIV-1 serology performed at the study site, of which 151 were confirmed by positive HIV-1 Western blot at University of Washington (Table 1). The analysis routine we developed is shown in Figure 1, and a linkage determination flow diagram for the pairs evaluated is shown in Supplementary Figure S1.

Of the 151 confirmed infections, 108 (71.5%) transmissions were classified as linked to the HIV-1 infected partner. Linkage determination was based on consensus HIV-1 *env* sequence data for 91 (84.3%), consensus *gag* sequences for 9 (8.3%), and from sequencing multiple clones or single molecule-derived amplicons (SM) *env* for 8 (7.4%). Forty transmissions (26.5%) were found to be unlinked and 3 (2.0%) had indeterminate linkage. Overall, 20 linked and 3 unlinked pairs had successful PCR amplification from only one gene. Of note, we were able to sequence both genes in all three HIV-1 infected participants (Partners 85A, 122A, and 132A) who reported antiretroviral therapy use before their partners seroconverted. Table 1 summarizes final linkage status by site, with sequence data for each pair and local control participants (LC) summarized in Supplementary Table S1. Sequences were submitted to GenBank (accession numbers HQ423670-HQ424010 and JF293469-JF294997). Phylogenetic trees are available at (http://www.mullinslab.microbiol.washington.edu/publications/campbell_2010).

### Phylogenetic analysis

**Figure 2:**
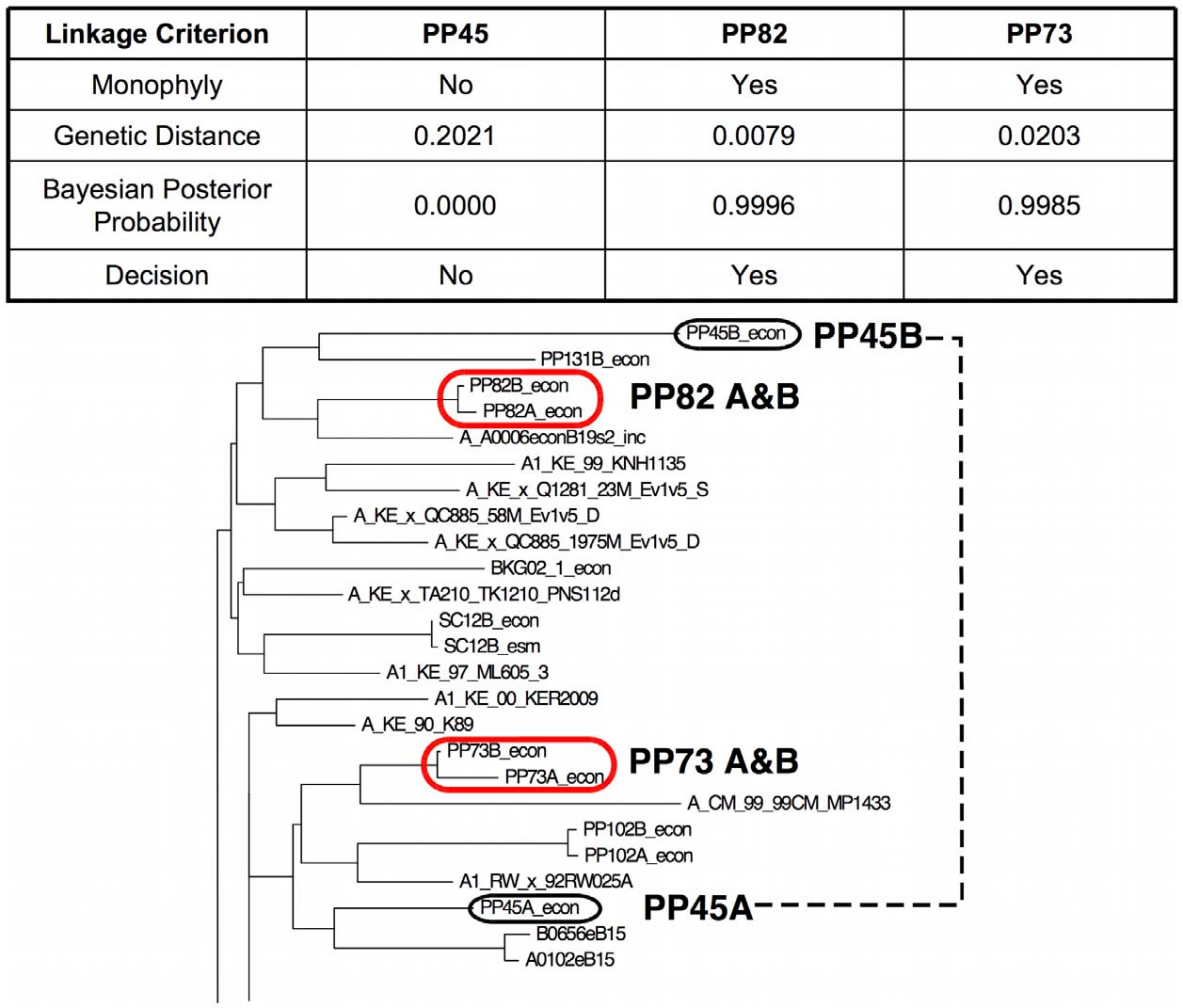
Examples of Phylogenetically Linked and Unlinked Transmission Events. A section of a phylogenetic tree showing examples of linked monophyletic (PP73 and PP82) and unlinked polyphyletic (PP45) pairs are shown, along with the adjudication criteria for each.

Among the 151 pairs, monophyly, defined as uniquely sharing a most recent common ancestor (MRCA) on the tree, was found for 84 pairs (55.6%) in both *env* and *gag,* but in *env* only for 16 pairs (10.6%), and in *gag* only for 9 (6.0%) pairs (Table S1). For the 25 pairs with linkage in only one gene, phylogenetic discordance between *env* and *gag* was found in only 4 partner pairs (PP135, SC2, PP133, and PP92) as only one gene was successfully amplified in the other 21 linked cases. Including the “local controls” (HIV-1 infected individuals from the same clinical site but not involved in a transmission event), we obtained sequences from a median of 17 individuals (range 10–80) at each study site. No local control sequence was found to split a monophyletic linkage between enrolled partners. Hence, linkages were unlikely to be erroneously assigned due to similarity to the circulating HIV-1 strains at each site. Figure 2 shows examples of monophyletic and polyphyletic partner pairs and the corresponding distance and Bayesian posterior probability data used for linkage adjudication.

### Genetic distance and Bayesian analysis

**Figure 3:**
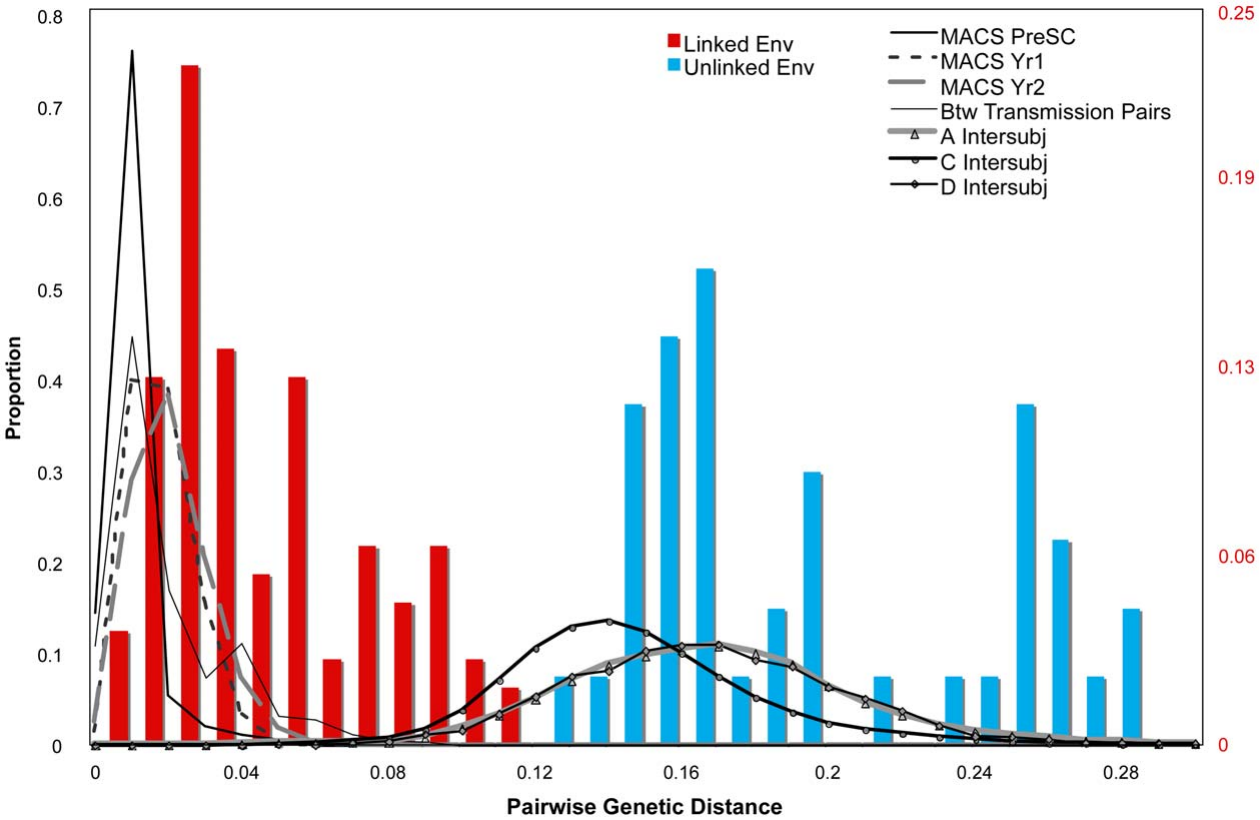
Pairwise Genetic Distances for *env.* Distributions of pairwise genetic distances for *env* reference datasets, within acutely infected individuals from the Multicenter AIDS Cohort Study at different intervals post infection, between epidemiologically-unlinked pairs of sequences from the HIVDB of subtypes A, C, and D (lines) and between enrolled partner-pairs from the Partners in Prevention HSV/HIV Transmission Study cohort that were adjudicated as linked (red bars) and unlinked (blue bars) through sequencing of *env*, *gag*, or both. To improve visibility of the data, the y-axis scale ranges from 0 to 0.25 for bars representing the Partners in Prevention HSV/HIV Transmission Study cohort.

**Figure 4:**
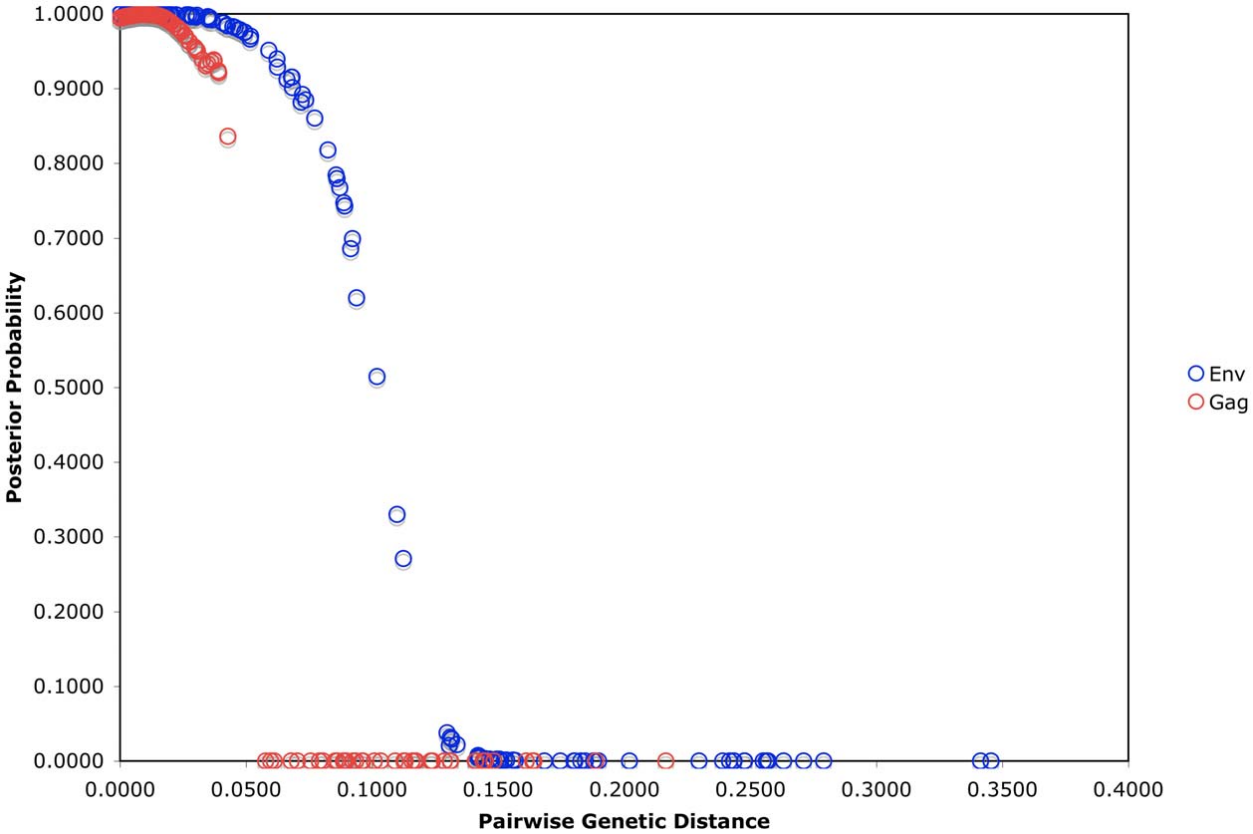
Bayesian Posterior Probabilities for *env* and *gag* Datasets. Plot showing relationship between Bayesian posterior probabilities and genetic distance between partner pairs from the Partners in Prevention HSV/HIV Transmission Study cohort in *env* and *gag*.

The median pairwise genetic distance for linked pairs was 2.8% (range 0.0–13.0%) in *env* and 1.3% (range 0.0–9.2%) in *gag* (Table 2). In unlinked pairs, median distances were 17.2% (range 11.2–34.6%) and 11.3% (range 6.0–21.6%) in *env* and *gag,* respectively. Distance ranges for linked and unlinked pairs overlapped due to pairs in which linkage was found in only one gene, *i.e.*, ranges for the linked pairs included distances for the gene which was not found to be linked in four cases (PP135, SC2, PP133, and PP92). Two of the three indeterminate pairs had genetic distances within the range of partner pairs that were linked in *env* (PP92) and *gag* (PP4 and PP92). However, only one indeterminate pair (PP92) exceeded the ≥50% posterior Bayesian probability cutoff, and in *env* only. Figure 3 shows the distribution of *env* genetic distances for linked and unlinked study pairs superimposed on the genetic distance distribution for intrasubject and intersubject linked and unlinked reference data (analogous data for *gag* sequences are shown in Supplementary Figure S3). Median Bayesian posterior probabilities for linked and unlinked pairs were 99.8% and 1.0% in *env* and 99.7% and 0.0% in *gag*, respectively. 97.2% and 98.1% of linked pairs met the Bayesian posterior probability cutoff of 50% in *env* and *gag*, respectively, while no unlinked pairs met this criterion (Table 2). As pairwise distance between couples' sequences increased, the Bayesian posterior probability of linkage decreased rapidly, with the majority of couples' pairwise distances associated with posterior probabilities approaching 1 (100% probability of linkage) or 0 (0% probability of linkage) (Figure 4).

**Table 2:** Comparison of monophyly, genetic distance, and Bayesian posterior probability results by linkage status and gene.

Criterion	Linked (N = 108)		Unlinked (N = 40)		Indeterminate (N = 3)	
	*env*	*gag*	*env*	*gag*	*env*	*gag*
Proportion with Monophyly	0.935	0.880	0	0	0.667	0
Median Pairwise Genetic Distance (range)	0.028(0.000–0.130)	0.013(0.000–0.092)	0.172(0.112–0.346)	0.113(0.060–0.216)	0.188(0.089–0.255)	0.079(0.068–0.161)
Median Bayesian Posterior Probability (range)	0.998(0.038–1.000)	0.997(0.000–1.000)	<0.001(0.000–0.271)	0.000(0.000–0.000)	0.000(0.000–0.743)	0.000(0.000–0.000)
Proportion with Bayesian Posterior Probability ≥0.50	0.972	0.981	0	0	0.333	0

* Monophyly is defined as sharing the most recent common ancestor on a phylogenetic tree. During evaluation, the adjudicators used the minimum pairwise genetic distance found between partners’ sequences within each couple and the corresponding Bayesian posterior probability for those most closely related sequence pairs. The median (range) for genetic distances and Bayesian posterior probabilities displayed in the table reflect the minimum genetic distance and maximum Bayesian posterior probability for each pair.

In two instances which were adjudicated as linked (PP47 and SC1), sequence pairs were monophyletic in *env* and *gag*, but with posterior probabilities <50% for *env* (45.8% and 33.0%) but high for *gag* (99.4% and 99.9%). In *gag*, no monophyletic pairs had posterior probabilities in an intermediate range (Figure 4 and Supplementary Table S1). Another pair (SC6) had sequences that were monophyletic in *gag* with a pairwise genetic distance of 5.8%, but with a Bayesian posterior probability of ∼0%. However, since SC6's *env* sequences met all criteria for linkage, it was classified as linked. In only one instance (SC2) did Bayesian analysis suggest linkage (posterior probability of 51.5%) in the absence of monophyly in the same gene (*env*). Because *gag* analysis met both phylogenetic and Bayesian criteria for linkage, this pair was also classified as linked.

### Deep sequencing (SM and pyrosequencing) for linkage determination

**Figure 5:**
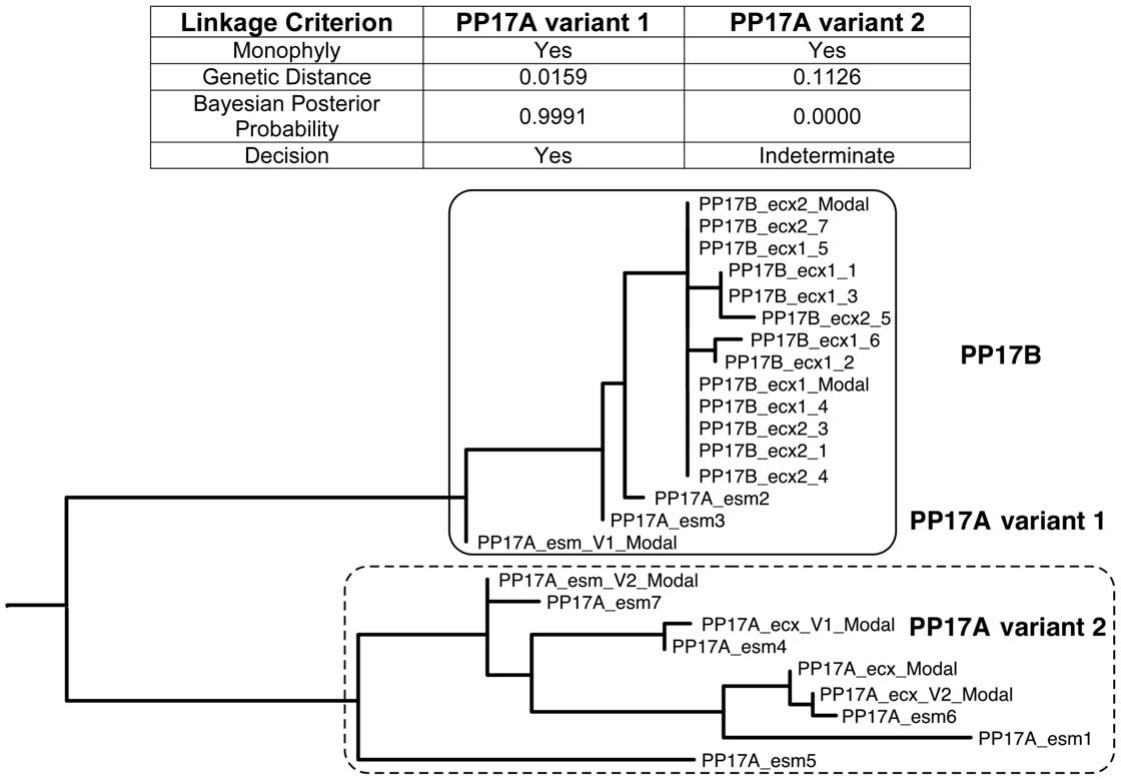
Examples of HIV-1 Transmission Classified as Linked by SM Sequencing. Example of a pair (Pair 17) whose consensus *env* sequences were unlinked, with linkage subsequently determined by single molecule (SM) *env* sequences. The linkage criteria used during adjudication are displayed in the table. Three linked sequences from the HIV-1 infected partner, PP17A variant 1, along with the sequences from the seroconverting partner PP17B are bounded by the solid rectangle. Unlinked sequences from the HIV-1 infected partner, PP17A variant 2 are delineated by the dotted rectangle.

We evaluated clonal or single molecule (SM) *env* sequences in 42 pairs that were unlinked or indeterminate by consensus sequencing with a median of 19 sequences evaluated per HIV-1 infected participant (range 3–62). Linkage was found in 8 (18.6%), with linked variants constituting 25–50% of the sequences evaluated for each linked pair. An example of the use of SM sequencing to establish linkage for a case (PP17) in which consensus sequences from the HIV-1 infected and seroconverting participants were unlinked is shown in Figure 5. In this case, 3 sequences from the HIV-1 infected partner had distances and Bayesian posterior probabilities that were categorized as linked to the seroconverter, whereas 9 other sequences did not meet this criterion. No relationship was found between classification of a pair as linked and the number of SM *env* sequences obtained.

When sufficient numbers of amplifiable viral templates (N = ∼50) were available for study, deep resequencing by pyrosequencing was used to probe for low-level variants. In 11 of 12 unlinked HIV-1 infected partners evaluated, involving a median of 119 templates per participant, we failed to detect sequences closely related to that in the seroconverter (Supplementary Table S2 and Figure S4). In the remaining case (PP9), 3.8% of the sequence reads from ∼61 viral templates from the HIV-1 infected case were closely related to viruses found in the seroconverter (Supplementary Table S1).

### Viral subtype

HIV-1 subtype was determined for both *env* and *gag* sequences (Tables 1 and S1) from each partner pair. In both genes, participants' viruses were predominantly subtype A or C (43% each in *env*, 44% and 36% in *gag*, respectively), with 13% of the *env* sequences and 10% of the *gag* sequences found to be subtype D. One subtype G infected pair was detected, and 2% of the *env* and 10% of the *gag* sequences were intersubtype recombinants. Among the 128 partner pairs with sequences determined for both *env* and *gag*, 13 pairs (10.1%) had different subtypes in *env* compared to *gag* genes, suggesting the presence of additional intersubtype recombinant viruses. In an additional 13 couples (4 linked, 6 unlinked, and 3 indeterminate pairs) discordant subtypes were identified between *env* and *gag* sequences in one partner, without such a discrepancy in the other partner.

When stratified by subtype, no statistically significant difference in the frequency of linked and unlinked pairs was found. Specifically, among linked transmissions, 69.8%, 66.7%, and 73.7% of *env* sequences, were subtype A, C, and D, respectively. The proportions of each subtype among linked transmissions were almost identical for *gag* sequences. Among intersubtype recombinants, 84.6% (11/13) were classified as linked.

### Discordant linkage findings

Eighty-four (95.5%) linked pairs having *env* and *gag* sequences met criteria for linkage in both genes. Among those classified as linked, two (2.3%) pairs met criteria for linkage in *gag* only (PP135 and SC2); and one (1.1%) pair met criteria for linkage in *env* only (PP133). The seroconverters in these 3 couples may have been infected by more than one HIV-1 strain. Eleven (7%) pairs had *env* and *gag* sequences of different subtypes, 2 from Zambia, 5 from Kenya and 4 from Uganda. Of these, 4 were classified as linked, with concordant *env* and *gag* subtypes between partners, suggesting that a virus with a recombinant subtype may have been transmitted from the HIV-1 infected partner to the seroconverter. In the remaining 7 pairs, 1 indeterminate and 6 unlinked, each partner's virus had a different mosaic subtype pattern.

### Adjudicator agreement and indeterminate pairs

During the 3 interim meetings, 3 HIV-1 sequencing experts reviewed available data and gave recommendations for pairs that required additional sequencing and analysis before their linkage status could be determined. At the end of the study the sequence data from all transmission pairs were reviewed by the adjudication committee. Complete agreement was reached between adjudicators' classification of all linked and unlinked pairs. Six (3.9%) pairs required discussion before all 3 adjudicators determined they were linked at the final adjudication meeting. Discussions focused on clarification of sequence labeling on phylogenetic trees and close inspection of phylogenies and pairwise distances in cases where deep sequencing had been performed. Adjudicators were unable to determine the linkage status of 3 pairs, described below, in which sequencing was completed (Supplementary Table S1).

Two pairs' data (PP4 and PP9) were suggestive of linkage in *env* only. PP4's consensus *env* and *gag* sequences were polyphyletic, with distances and Bayesian posterior probabilities outside the expected range for linked transmissions (Table S1). The viral subtype in *env* was C for the female HIV-1 infected participant and A for her male seroconverting partner. After SM *env* sequencing, 1 of 17 sequences from the HIV-1 infected participant was found to be of subtype A and fell in a monophyletic cluster with the seroconverter's sequences. However, the subtype A *env* sequence's pairwise genetic distance (18.8%) and Bayesian posterior probability (0.0%) were inconsistent with linkage, as was *gag* data, so Pair 4 was categorized as indeterminate.

Similarly, consensus *env* sequences from the female HIV-1 infected and male seroconverting partners of PP9 were of different subtypes (C and A, respectively). Both consensus *env* and *gag* sequences were polyphyletic and with large distances (25.5 and 16.1%, respectively). SM *env* sequencing from both participants (N = 16 and 29, respectively) did not reveal any more closely related sequences. However, approximately 61 *env* templates from the HIV-1 infected participant were subjected to pyrosequencing, which did reveal a variant that was closely related to the seroconverting partner's virus, comprising 3.8% of the viral population on the 3′ ends of the amplicon, with no close relatives above the 100 nt cutoff read length from the 5′ end reads (4 short reads, corresponding to 0.2% of the total sequences were found to be related to the seroconverter consensus but were discarded due to poor quality). The adjudication team concluded that the small fraction of related sequences found by a sequencing technique that is still in development for applications related to HIV-1 evolution did not provide sufficient evidence to categorize this pair as linked.

Finally, PP92's consensus *env* sequences were monophyletic, but with a large pairwise distance of 8.9%. After consensus *gag* sequences were found to be polyphyletic and relatively distant (6.8%) and 17 SM *env* sequences from the HIV-1 infected partner did not reveal a sequence with a smaller genetic distance to the serooconverter's virus, this pair was also classified as indeterminate.

### Epidemiologic support for linkage assignments

We compared demographic and clinical characteristics of linked and unlinked partnerships to examine their associations with linkage (Table 3). The seroconverting partner was male in 88 (58.3%) and female in 63 (41.7%) of the 151 couples, reflecting, in part, the study enrollment gender distribution with 67% of enrolled partners being male. However, seroconverters were female in a larger proportion of linked relative to unlinked pairs (46.3% versus 27.5%, p = 0.04). The timing of seroconversion also was associated with linkage, with linked pairs having a shorter average time to seroconversion than unlinked pairs (6 versus 12 months after enrollment, p = 0.001). Furthermore, there was a trend toward the proportion of linked transmissions being greater among seroconversions identified at the first 3-month study visit compared to seroconversions identified after 3 months (89.5% versus 66.9%, p = 0.06). Sexual activity with the HIV-1 infected partner at the 3-month study visit prior to seroconversion was reported more commonly by linked than unlinked seroconverters (87% versus 70%, p = 0.027). Conversely, sex with partners other than the HIV-infected partner with whom they enrolled was reported more commonly by unlinked than linked seroconverters (30% versus 1.9%, p<0.001) and the majority of these unlinked partners were male. Finally, baseline plasma HIV-1 RNA levels for the HIV-1 infected partner were higher among linked pairs than unlinked pairs (4.7 versus 4.0 log_10_ copies/ml, p<0.001).

**Table 3:** Association of demographic and clinical factors with linkage.

	All Pairs (N = 155)	Linked Pairs (N = 108)	Unlinked Pairs (N = 40)	p value^*^
**Gender**				
Seroconverting partner female	64 (41.3%)	50 (46.3%)	11 (27.5%)	0.041
**Median age at enrollment (range)**				
Seroconverting partner	30 (26–38)	30 (25–38)	32 (26–36)	0.425
HIV-1 infected partner	31 (25–37)	30.5 (26–36)	31 (25–39.5)	0.935
**Time to seroconversion**				
Identified at 3 month visit	19 (12.3%)	17 (15.7%)	2 (5.0%)	0.101
Months of follow-up before seroconversion	9 (3–15)	6 (3–15)	12 (9–17)	0.001
**Study site location**				
East Africa	96 (61.9%)	68 (63.0%)	23 (57.5%)	0.572
Southern Africa	59 (38.1%)	40 (37.0%)	17 (42.4%)	0.572
**Behavioral characteristics of seroconverting partner prior to seroconversion**				
Reported sex with HIV-1 infected partner^t^	127 (81.9%)	94 (87.0%)	28 (70.0%)	0.027
Reported sex with a non-enrolled partner^t^	14 (9.0%)	2 (1.9%)	12 (30.0%)	<0.001
Female	3 (4.7%)	1 (2.0%)	2 (18.2%)	0.081
Male	11 (12.1%)	1 (1.7%)	10 (34.5%)	<0.001
**Characteristics of HIV-1 infected partner**				
Enrollment plasma HIV-1 RNA (log_10_ copies/mL)	4.6 (4.0–5.1)	4.7 (4.3–5.1)	4.0 (3.5–4.8)	<0.001
CD4 count (cells/uL) at visit closest to seroconversion	379 (281–506)	364 (255-495)	369 (307-502)	0.323
Reported use of antiretroviral therapy at visit prior to seroconversion	3 (1.9%)	1 (0.9%)	2 (5.0%)	0.178

Comparison of linked and unlinked transmission pairs.

t Calculated for each 3 month period of observation.

## Discussion

We conducted an evaluation of HIV-1 transmission linkage by analysis of phylogenetic and genetic distance data and Bayesian posterior probabilities among HIV-1 seroconverters who were followed prospectively in a cohort of east and southern African HIV-1 serodiscordant couples. Through a hierarchical, multi-step process based on sequencing, phylogenetic and Bayesian statistical analysis, and independent adjudication, we found that over one quarter (26.5%) of HIV-1 transmission events within this cohort were not linked to the enrolled partner.

Numerous studies have used viral sequencing to evaluate HIV-1 transmission linkage, but our analysis represents the first use of viral sequencing for HIV-1 transmission linkage as an integral component in the primary efficacy analysis of a large randomized HIV-1 prevention trial. Because the trial's intervention was intended to reduce infectiousness in the HIV-1 infected partner, only linked transmissions were relevant to ascertainment its efficacy. As with previous linkage assessments in observational studies [35], our protocol (Figure 1 and Table 4) included an evaluation of sequence data from *env* and *gag* for monophyly in maximum likelihood trees to determine linkage. However, to provide additional statistical support for our linkage determinations, we developed a Bayesian algorithm incorporating prior probability of linkage and genetic distance data and increased our sensitivity for detecting rare variants in the HIV-1 infected partner that may have been transmitted to the seroconverter using deep sequencing techniques. While consensus *env* sequencing identified 85% of linked pairs, *gag* and deep *env* sequencing permitted classification of an additional 9 (8.3%) and 8 (7.4%) linked pairs, respectively, that would not have been linked if only consensus *env* were used to define linkage. Our Bayesian algorithm provides a quantitative assessment of linkage and offers additional perspective for the genetic distance data, by relating those data to the expected distance ranges for linked and unlinked sequence pairs. It did not, however, take precedence over phylogenetic linkage determinations. In 3 pairs (PP47, SC1, and SC6), linkage decisions were based on monophyly despite having Bayesian posterior probabilities <50%. In future studies, if a particular site lacked ”local controls” or other geographically-appropriate reference sequences, the Bayesian algorithm could be helpful in differentiating monophyletic pairs associated with transmission versus those that were more genetically distant, yet clustered due to geographic location. The determination of linkage by individual adjudicators was highly consistent, with identical independent assessments in 96% of cases, followed by full concurrence after discussion. If only one scientist had evaluated the data, 6 (5.6%) of the linked pairs may have received an indeterminate designation, which suggests that discussion amongst experts during the adjudication process was helpful in resolving uncertainties in interpretation of the data. In only 3 (2.0%) of cases were adjudicators unable to determine the linkage status conclusively, possibly due to HIV-1 dual or superinfection followed by recombination of viral strains. Additional deep or whole genome sequencing may resolve such indeterminate classifications, but was beyond the scope of this study.

**Table 4:** Summary of sequencing and analysis methods used in this study.

	Benefits	Limitations	Impact on this study
**Data type**			
Consensus *env*	-C2-V3-C3 region captures variation in *env* and lacks large insertions and deletions found in other regions of the gene-Large number of sequences in HIVDB	-Only reveals variant that comprises majority of plasma viruses	-84.3% (91/108) pairs linked by consensus *env*
Consensus *gag*	-p17 and p24 regions contain variable areas within *gag*-Longer sequence than consensus *env*	-Only reveals variant that comprises majority of plasma viruses-Less variable than *env* and fewer published sequences in HIVDB	-8.3% (9/108) additional pairs linked only by consensus *gag*
Single molecule (SM) *env*	-Improves sampling of circulating plasma viruses to better capture variants within an individual	-Requires more laboratory effort than consensus sequencing	-Performed for 27.8% (42/151) of pairs that were unlinked or indeterminate by consensus sequencing, with 7.4% (8/108) additional pairs linked only by SM *env*
*env* pyrosequencing	-Facilitates sampling larger number of viruses compared to SM	-Computational challenges in data analysis	-Performed for 7.9% (12/151) of pairs that were unlinked by consensus, but did not lead to more pairs linked and adjudicators questioned reliability of data
**Analysis method**			
Phylogenetic tree	-Shows relationships between multiple sequences	-Relationships between sequences are contextual and depend upon the relatedness of all sequences in the phylogeny, i.e., additional “local control” sequences are valuable as they can disrupt fortuitous monophyletic relationships	-Sequences from all linked pairs were monophyletic in *env*, *gag*, or both and sequences from unlinked pairs were polyphyletic in both genes-Indeterminate pairs had suggestion of linkage in *env*, but not in *gag*
Pairwise genetic distance	-Numerical result that compares the differences between each nucleotide in 2 sequences	-Viruses within transmitters can have wide genetic variation and thus it is not possible to set an *a priori* cutoff value for linkage	-Clear difference in median distances in linked and unlinked pairs, but distance ranges overlapped due to pairs that were linked in 1 gene but not the other
Bayesian posterior probability	-Estimates the probability that the genetic distance between sequences from a putative HIV-1 transmission pair represents a within-couple transmission	-Relies on reference data for epidemiologically linked and unlinked HIV-1 infected individuals	-Median posterior probabilities for linked and unlinked pairs approached 100% and 0%, respectively-Posterior probability ≥50% in only 1 pair whose *env* sequences were polyphyletic
Expert adjudication	-Provides a forum for resolving uncertainties in data interpretation-Reduces potential for subjectivity and error in linkage determination	-Requires multiple scientists’ time/effort	-Discussions at interim meetings guided further sequencing efforts-Experts agreed on all final classifications of linked and unlinked pairs and were unable to classify only 2% (3/151) of pairs

The majority of linked and unlinked pairs were clearly separated by phylogenetic relationships, genetic distance, and Bayesian posterior probability estimates, allowing adjudicators to definitively classify transmission linkage for 98% (148/151) of putative transmission events. Our finding that ∼27% of seroconverters' HIV-1 sequences were unlinked to those of their enrolled partners underscores that transmission linkage cannot be assumed, and in doing so, provides a guide to help minimize uncertainty in HIV-1 transmission linkage assignment for future observational studies of HIV-1 infectiousness and trials of candidate prevention interventions to reduce HIV-1 infectiousness.

Nevertheless, several limitations are noteworthy. First, we could not determine the relative utility of each type of sequencing in linkage determination because our protocol did not require all three types of sequencing data from each individual, but we advocate for use of consensus *env* sequencing at a minimum, as 85% of linked pairs were determined by analysis of partial consensus *env* sequences, followed by consensus *gag* and multiple *env* variant sequencing, as needed. Second, our ability to sequence “local controls” from each study site was also limited. Ideally, we would have sequenced a robust sample of the circulating viral population from epidemiologically unlinked individuals that corresponded to the HIV-1 subtype found in participants at each study site. However, when we received participants' plasma specimens, the viral subtypes were unknown and the clinical trial's time constraints precluded sequencing from additional unlinked participants. We found that the subtype A, C, and D sequences from Africa retrieved from the HIVDB adequately separated the sequences from sites in our study, minimizing the possibility that geographic clustering led to false evidence of linkage. Finally, the epidemiologically linked reference sequence data sets used to develop the Bayesian algorithm were not taken exclusively from transmission pairs whose HIV-1 risk factor was heterosexual sex. There were few publicly available sequences from genetically linked heterosexual HIV-1 transmissions and we therefore needed to include data from male-male and mother-infant transmission pairs. However, as the trial proceeded, sequence pairs that the adjudicators determined to be linked were added to this database, such that the data from linked pairs in our trial outnumbered those from published reference sets.

Recent data has revealed the role of minority viral variants from the transmitting partner in individuals acquiring HIV-1 through heterosexual sex [22], [38], [39], which was our rationale for using deep sequencing techniques in couples whose viruses were initially found to be unlinked. An additional step that could have been performed was analysis of HIV-1 sequences from genital specimens of HIV-1 infected partners. Although it is theoretically possible that sequencing from blood may have missed a viral variant present exclusively in a genital compartment, the likelihood of this is low, as analyses of seminal and cervicovaginal specimens have shown that viral sequences from blood and genital sites cluster monophyletically and often intermingle within an individual [40]–[42].

The relatively high fraction (26.5%) of unlinked infections we found differs from a cohort study of HIV-1 serodiscordant couples in Zambia from 1994-2000, in which 13% of prospectively identified seroconverters were found to have viruses not linked to their stable partner [35]. Their study used analogous laboratory methods, involving amplification of HIV-1 *env* and *gag* consensus sequences from blood plasma RNA, and similarly evaluated phylogenetic relationships and genetic distances. While the two studies cannot be directly compared due to differences in design, location, and period of conduct, it is notable that a greater proportion of HIV-1 infected partners in the Zambian cohort were male compared to our cohort (52% versus 33%). In our study, male seroconverters were significantly more likely than females to report sex with additional partners; it is plausible that the greater proportion of male seroconverters with unlinked viruses is a consequence of this behavior and may explain the higher rate of unlinked infections our cohort. Our finding of more unlinked seroconversions occurring later after study enrollment most likely is related to the increase in reported sexual activity with partners other than those with whom they were enrolled during the 2 years of follow-up [14]. The strong associations we saw between unlinked transmission and reported sexual activity with additional partners and the higher proportion of female seroconverters found to be infected from their stated partners, corroborates our linkage assignments and suggest that behavioral rather than biological factors may underlie the higher rate of non-linkage in our cohort. Our rigorous evaluation of transmission linkage reduced potential misclassification of over a quarter of seroconversion endpoints – a substantial issue for efficacy trials of interventions to reduce HIV-1 infectiousness. Insofar as the unlinked transmissions represent HIV-1 infection transmitted from outside the stable HIV-1 serodiscordant partnership, our findings underscore the importance of incorporating messages that underscore the risk of sex with partners of unknown serostatus when working with HIV-1 serodiscordant couples. In addition, our finding of nearly 30% of HIV-1 transmissions being genetically unlinked and likely acquired from an outside partner in these African couples, indicates a need for biomedical interventions, such as vaccines, microbicides and pre-exposure prophylaxis, for the HIV-1 seronegative partner in serodiscordant partnerships.

The Partners in Prevention HSV/HIV Transmission Study did not find an association of HSV-2 suppression with a change in HIV-1 transmission through either a modified intent-to-treat analysis evaluating only linked HIV-1 transmission events, or a per-protocol-analysis that evaluated all eligible HIV-1 transmission events [14]. However, for future studies, the erroneous assumption of linkage for one quarter of identified transmission events could clearly be a major source of misclassification bias with a consequent high risk of inaccurate conclusions about risk factors for HIV-1 transmission or efficacy of interventions to reduce HIV-1 infectiousness. While the need for deep sequencing in future HIV-1 transmission linkage confirmation algorithms bears further study, our findings suggest that analysis of HIV-1 sequences from two potentially linked individuals in a clinical trial should include: 1) consensus *env,* 2) consensus *gag,* 3) analysis for monophyly and pairwise genetic distance in both gene regions, 4) Bayesian posterior probability calculations incorporating the prior probability of linkage and the pairwise genetic distances, 5) discussion/adjudication by experts, and 6) sequencing of multiple *env* variants in putative transmitting partners in couples without clear evidence of linkage by consensus sequencing. Our approach is relevant both for future HIV-1 prevention trials evaluating interventions that target the HIV-1 infected partner and for studies seeking to characterize virologic, immunologic, and host genetic determinants of HIV-1 transmission.

## Supporting Information

Figure S1 **Overview of Laboratory and Analysis Methods**. (**a**) Overview of laboratory methods. RNA was extracted from blood plasma, cDNA synthesized, and multiplex PCR targeting *env* and *gag* was performed. Sequences were aligned and analyzed in the context of reference and ‘local control’ sequences of the same subtype. Phylogenetic relationships, pairwise genetic distances, and Bayesian posterior probabilities were obtained. (**b**) Process by which posterior probabilities of linkage were obtained. The linked dataset corresponded to sequences derived from the Los Alamos National Laboratory HIV database (HIVDB) and trimmed to match the amplicons sequenced in the current study in *env* and *gag*. The linked dataset was composed of intrasubject sequences from <2 years after infection from the MACS, from available linked partner pairs from the literature and intermediate adjudications in this study, and from mother-infant transmission pairs. Three unlinked datasets were initially derived, from HIV-1 subtypes A, B and C, one sequence per subject and from individuals with no known epidemiologic linkage. After each set of sequences were aligned, pairwise distances were determined and the each dataset combined to create one “linked’ and one “unlinked” pairwise distance dataset. Alignments are available at (http://www.mullinslab.microbiol.washington.edu/publications/campbell_2010). These datasets were used to estimate prior probabilities of linkage using the Bayesian approach described in Methods.
(PPT)

Figure S2 **Linkage** **Results** **Flow Chart.** Flow chart of sequences obtained and linkage results for all pairs evaluated. *Consensus *gag* sequence analysis contributed 5 linkages in eligible pairs and 4 linkages in 3-month seroconverters (circles) over consensus *env* sequencing alone. Deep sequencing by clonal or single molecule (SM) and amplicon pyrosequencing (pyro) of *env* revealed 8 additional linked pairs. Deep sequencing was not performed in 3-month seroconverter pairs, as they were not included in the modified intention to treat analysis.
(TIF)

Figure S3 **Pairwise Genetic Distances for Reference** ***gag*** **Datasets.** Distributions of pairwise genetic distances for *gag* reference datasets and between enrolled partner-pairs from the Partners in Prevention HSV/HIV Transmission Study cohort that were adjudicated as linked (red bars) and unlinked (blue bars) through sequencing of *env*, *gag*, or both.
(TIF)

Figure S4 **Pyrosequencing Analysis.** Each panel shows the distribution of pairwise genetic distances between a reference sequence (the consensus of *env* sequences from each seroconverting partner) and pyrosequences derived from the index partner. See Supplementary Table S2 for details. The graph on the left side of each panel shows the analysis of the 5′ and 3′ reads, respectively. Distributions marked in blue indicate the relationship of the HIV-1 infected partners' sequences to the consensus of the seroconverting partners' sequence. Distributions marked in red indicate the relationship of seroconverting partners' sequences to the consensus of the HIV-1 infected partners' sequence.
(PPT)

Table S1 **Summary of HIV-1 Transmission Linkage and Local Control Sequence Data.** Demographic, sequence, and linkage data for each couple and “local control” participant. Pairwise nucleotide distances shown are the smallest pairwise distances obtained, from either consensus or single molecule sequencing in *env* or consensus sequencing in *gag*, with Bayesian posterior probabilities corresponding to the distances shown.
(XLS)

Table S2 **Summary of HIV-1** ***env*** **Pyrosequencing Analysis.** Pyrosequencing analysis of the HIV-1 infected partner's *env* sequences in pairs of individuals without prior evidence of linkage. The approximate number of templates evaluated in each pyrosequencing reaction are shown, along with the number of raw and final reads used in the evaluation. 400 bp amplicons were sequenced using primers from the 5′ and 3′ ends. The ∼220 bp reads from each end were analyzed separately. A variable number of sequences were removed from the final alignments as described in the Methods. Pyrosequencing on the thirteenth pair listed, PP118, did not yield sequence data due to insufficient read length.
(TIFF)
