# Supplementary figures and images for: Viral Linkage in HIV-1 Seroconverters and Their Partners in an HIV-1 Prevention Clinical Trial
Source: PLoS One. 2011 Mar 2;6(3):e16986. doi: 10.1371/journal.pone.0016986 (PMC3047537; doi:10.1371/journal.pone.0016986)

## Slide 1
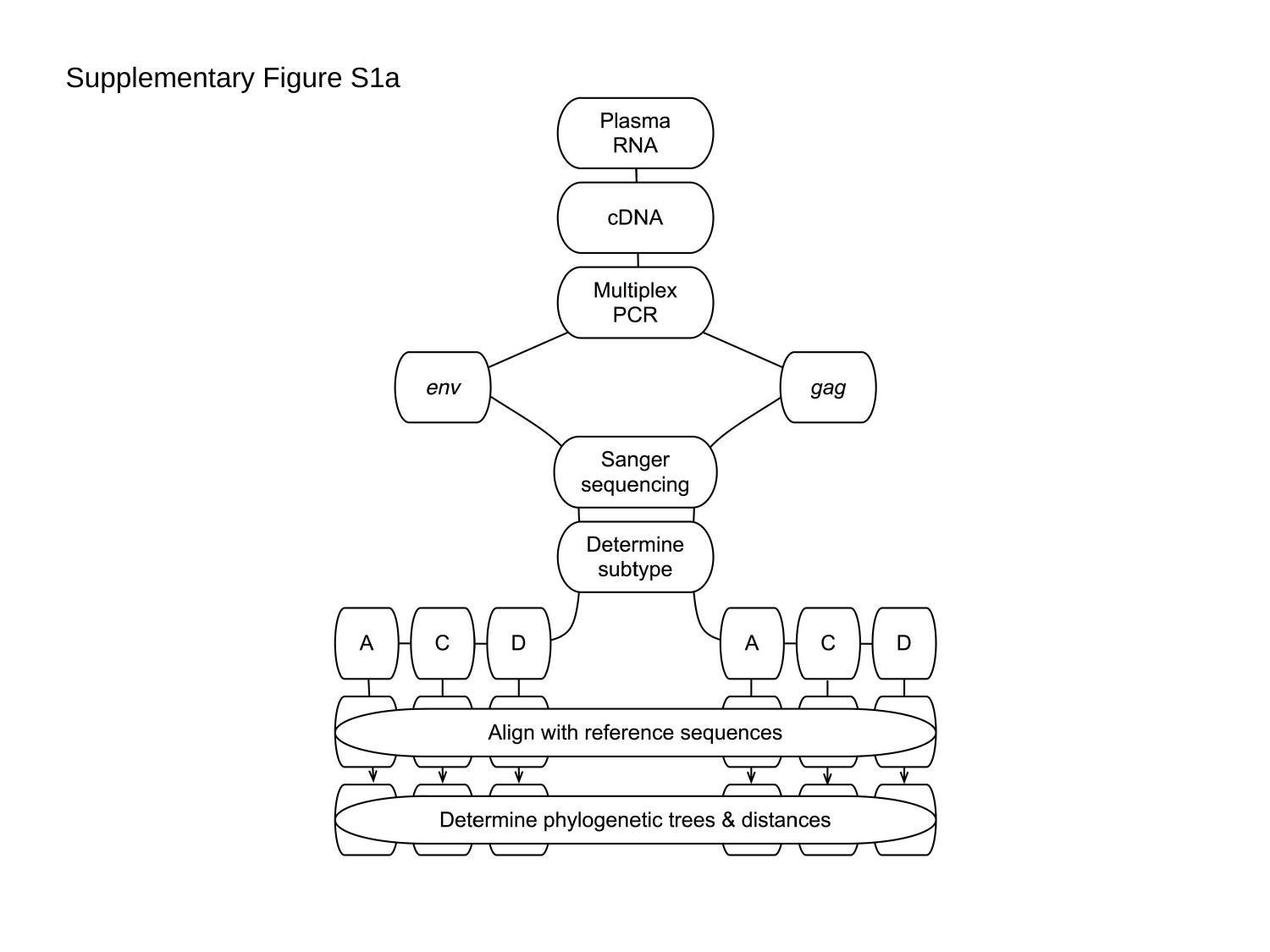

Supplementary Figure S1a

## Slide 2
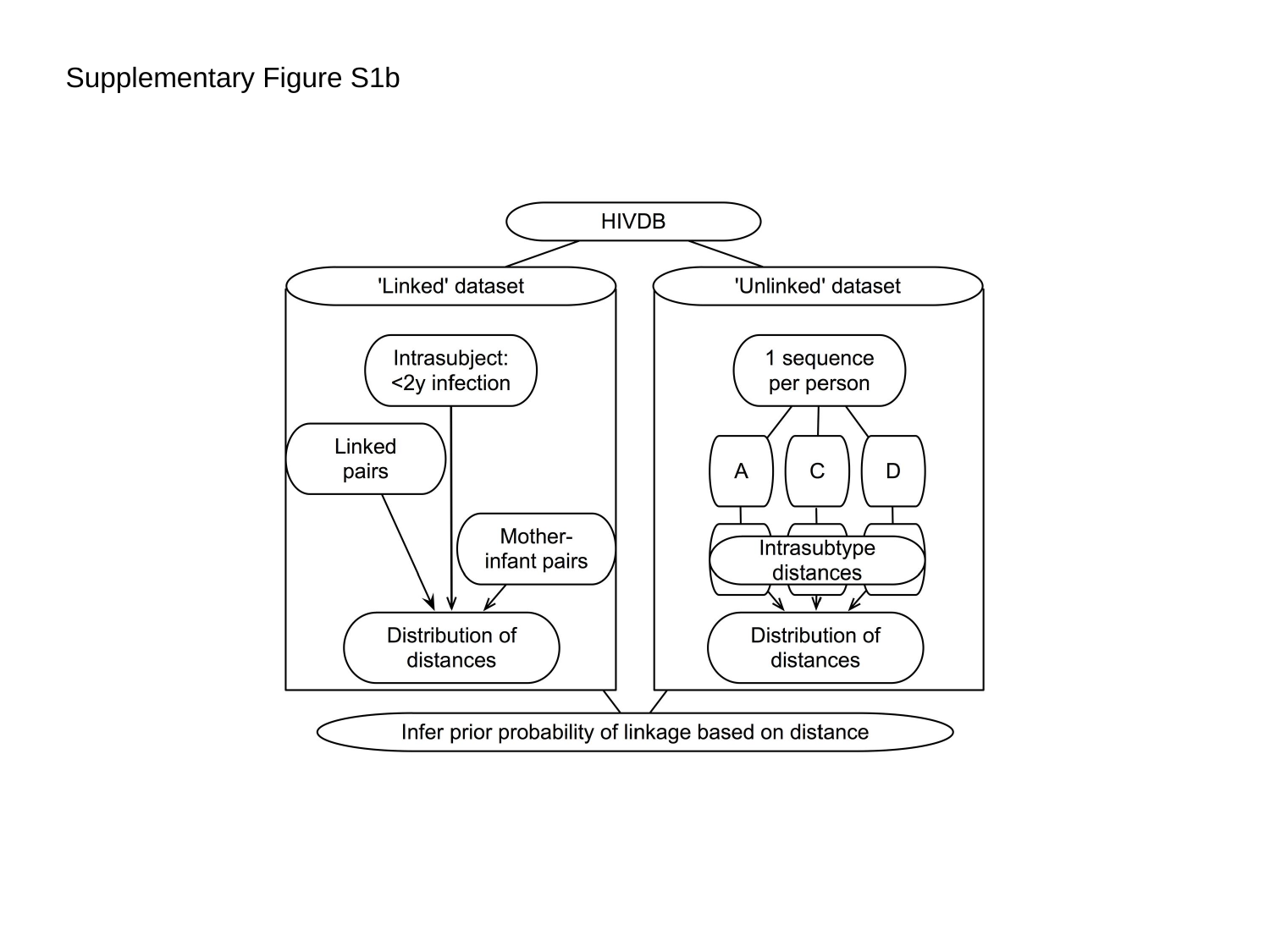

Supplementary Figure S1b

Supplement: Figure S1 — Overview of Laboratory and Analysis Methods. (a) Overview of laboratory methods. RNA was extracted from blood plasma, cDNA synthesized, and multiplex PCR targeting env and gag was performed. Sequences were aligned and analyzed in the context of reference and ‘local control’ sequences of the same subtype. Phylogenetic relationships, pairwise genetic distances, and Bayesian posterior probabilities were obtained. (b) Process by which posterior probabilities of linkage were obtained. The linked dataset corresponded to sequences derived from the Los Alamos National Laboratory HIV database (HIVDB) and trimmed to match the amplicons sequenced in the current study in env and gag. The linked dataset was composed of intrasubject sequences from <2 years after infection from the MACS, from available linked partner pairs from the literature and intermediate adjudications in this study, and from mother-infant transmission pairs. Three unlinked datasets were initially derived, from HIV-1 subtypes A, B and C, one sequence per subject and from individuals with no known epidemiologic linkage. After each set of sequences were aligned, pairwise distances were determined and the each dataset combined to create one “linked’ and one “unlinked” pairwise distance dataset. Alignments are available at (http://www.mullinslab.microbiol.washington.edu/publications/campbell_2010). These datasets were used to estimate prior probabilities of linkage using the Bayesian approach described in Methods. (PPT) [file pone.0016986.s001.ppt]

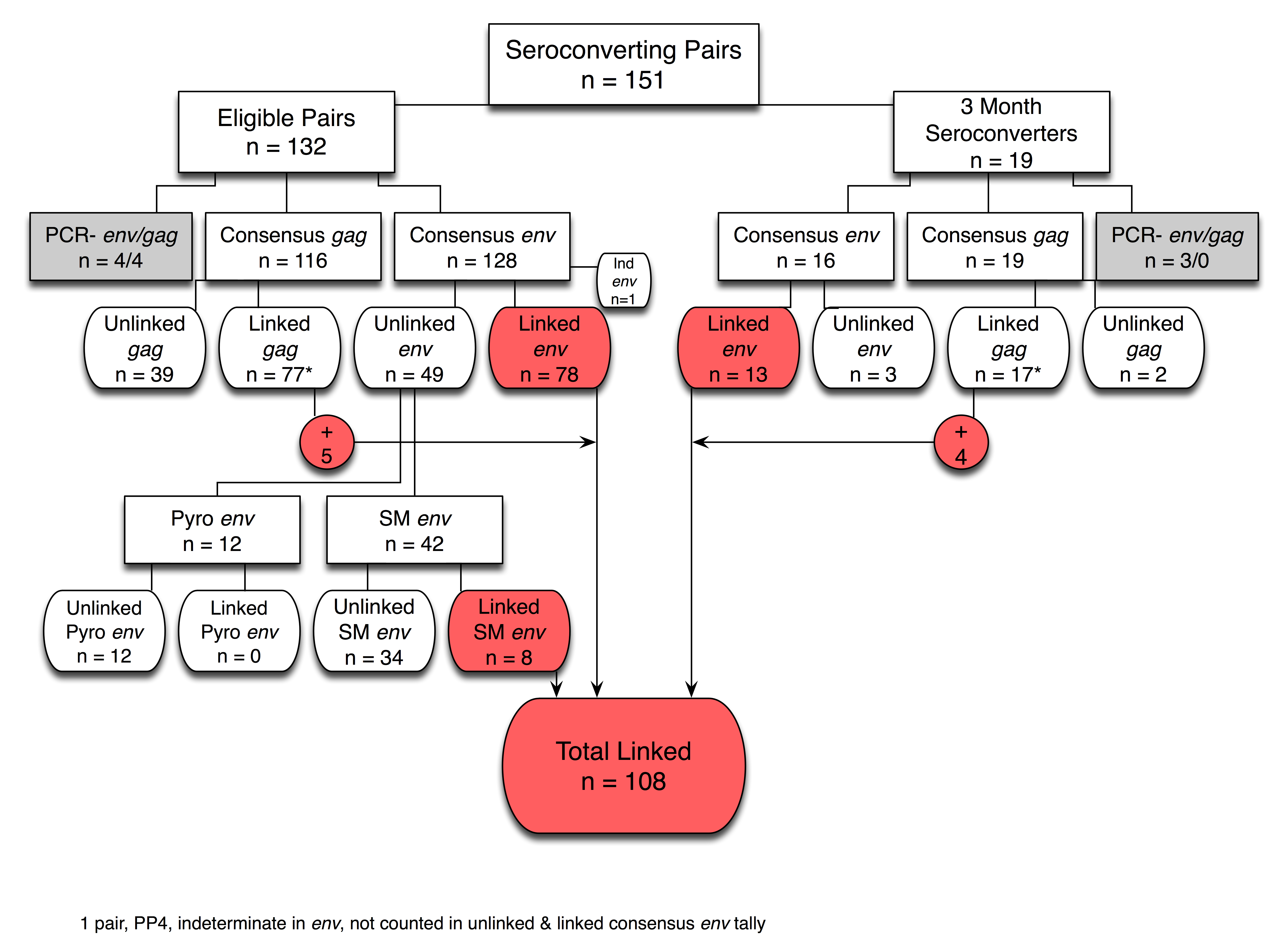

Supplement: Figure S2 — Linkage Results Flow Chart. Flow chart of sequences obtained and linkage results for all pairs evaluated. *Consensus gag sequence analysis contributed 5 linkages in eligible pairs and 4 linkages in 3-month seroconverters (circles) over consensus env sequencing alone. Deep sequencing by clonal or single molecule (SM) and amplicon pyrosequencing (pyro) of env revealed 8 additional linked pairs. Deep sequencing was not performed in 3-month seroconverter pairs, as they were not included in the modified intention to treat analysis. (TIF) [file pone.0016986.s002.tif]

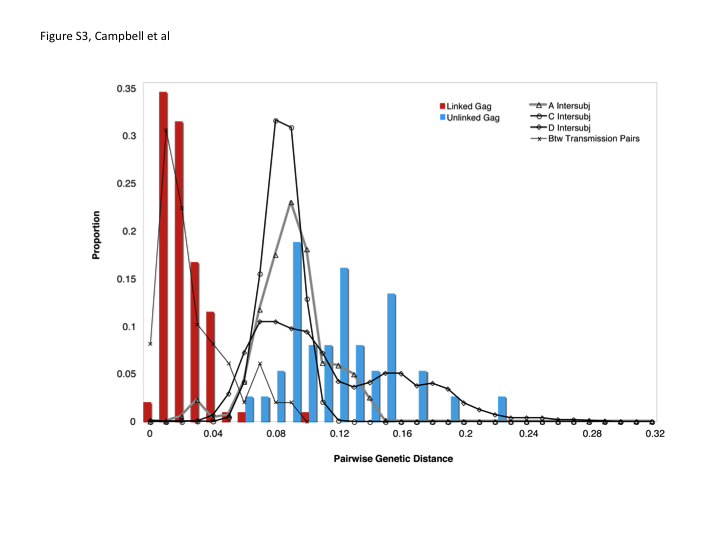

Supplement: Figure S3 — Pairwise Genetic Distances for Reference gag Datasets. Distributions of pairwise genetic distances for gag reference datasets and between enrolled partner-pairs from the Partners in Prevention HSV/HIV Transmission Study cohort that were adjudicated as linked (red bars) and unlinked (blue bars) through sequencing of env, gag, or both. (TIF) [file pone.0016986.s003.tif]

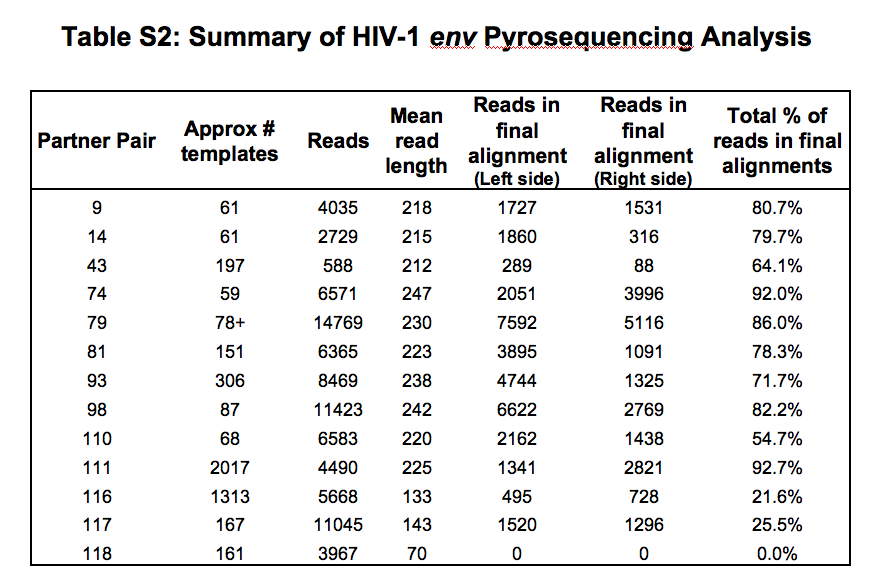

Supplement: Table S2 — Summary of HIV-1 env Pyrosequencing Analysis. Pyrosequencing analysis of the HIV-1 infected partner's env sequences in pairs of individuals without prior evidence of linkage. The approximate number of templates evaluated in each pyrosequencing reaction are shown, along with the number of raw and final reads used in the evaluation. 400 bp amplicons were sequenced using primers from the 5′ and 3′ ends. The ∼220 bp reads from each end were analyzed separately. A variable number of sequences were removed from the final alignments as described in the Methods. Pyrosequencing on the thirteenth pair listed, PP118, did not yield sequence data due to insufficient read length. (TIFF) [file pone.0016986.s006.tif]
