# Supplementary material for: Viral Linkage in HIV-1 Seroconverters and Their Partners in an HIV-1 Prevention Clinical Trial
Source: PLoS One. 2011 Mar 2;6(3):e16986. doi: 10.1371/journal.pone.0016986 (PMC3047537; doi:10.1371/journal.pone.0016986)
Supplement: Figure S4 — Pyrosequencing Analysis. Each panel shows the distribution of pairwise genetic distances between a reference sequence (the consensus of env sequences from each seroconverting partner) and pyrosequences derived from the index partner. See Supplementary Table S2 for details. The graph on the left side of each panel shows the analysis of the 5′ and 3′ reads, respectively. Distributions marked in blue indicate the relationship of the HIV-1 infected partners' sequences to the consensus of the seroconverting partners' sequence. Distributions marked in red indicate the relationship of seroconverting partners' sequences to the consensus of the HIV-1 infected partners' sequence. (PPT) [file pone.0016986.s004.ppt]

## Slide 1
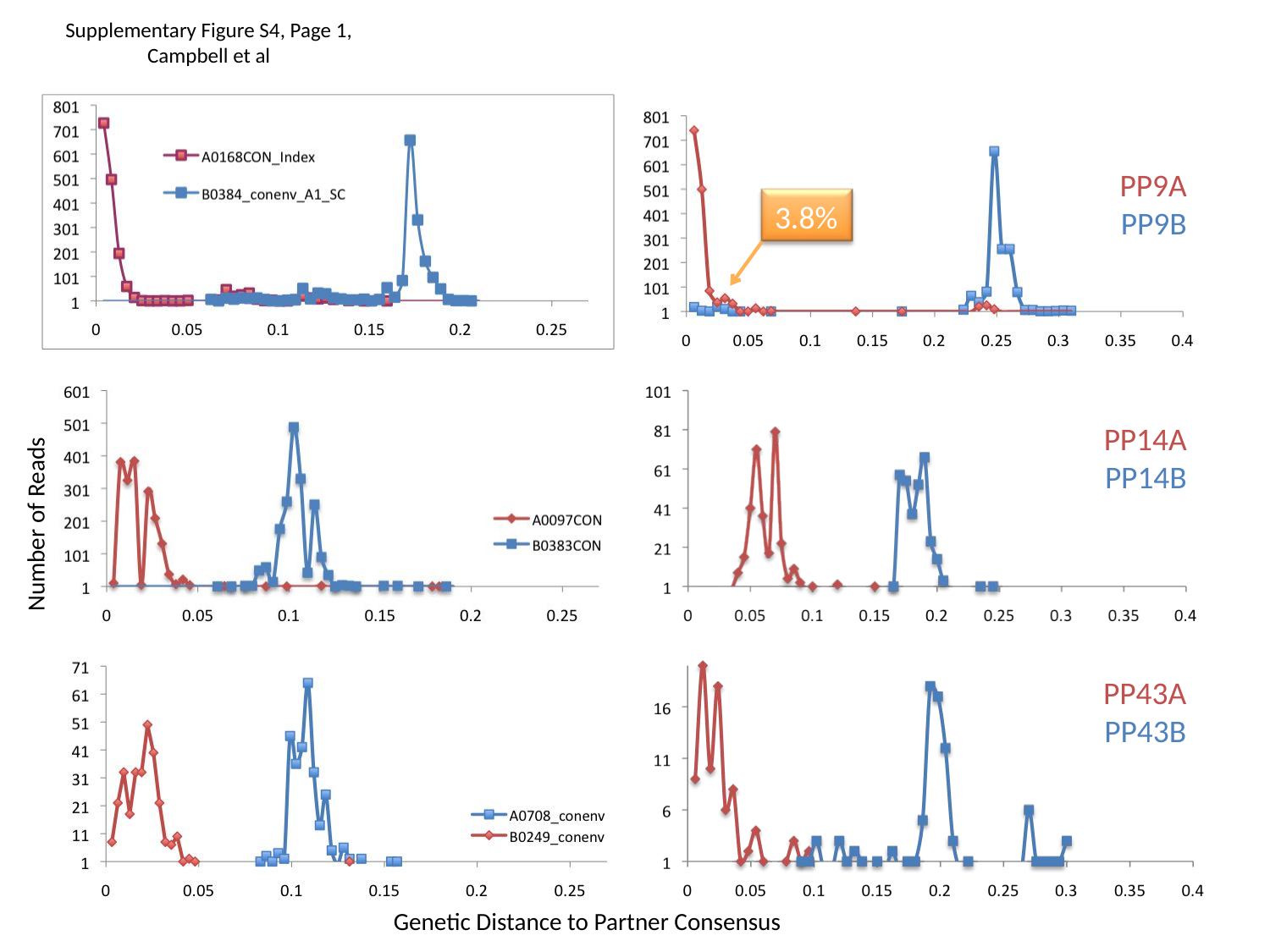

Supplementary Figure S4, Page 1, Campbell et al
PP9A
PP9B
3.8%
PP14A
PP14B
Number of Reads
PP43A
PP43B
Genetic Distance to Partner Consensus

## Slide 2
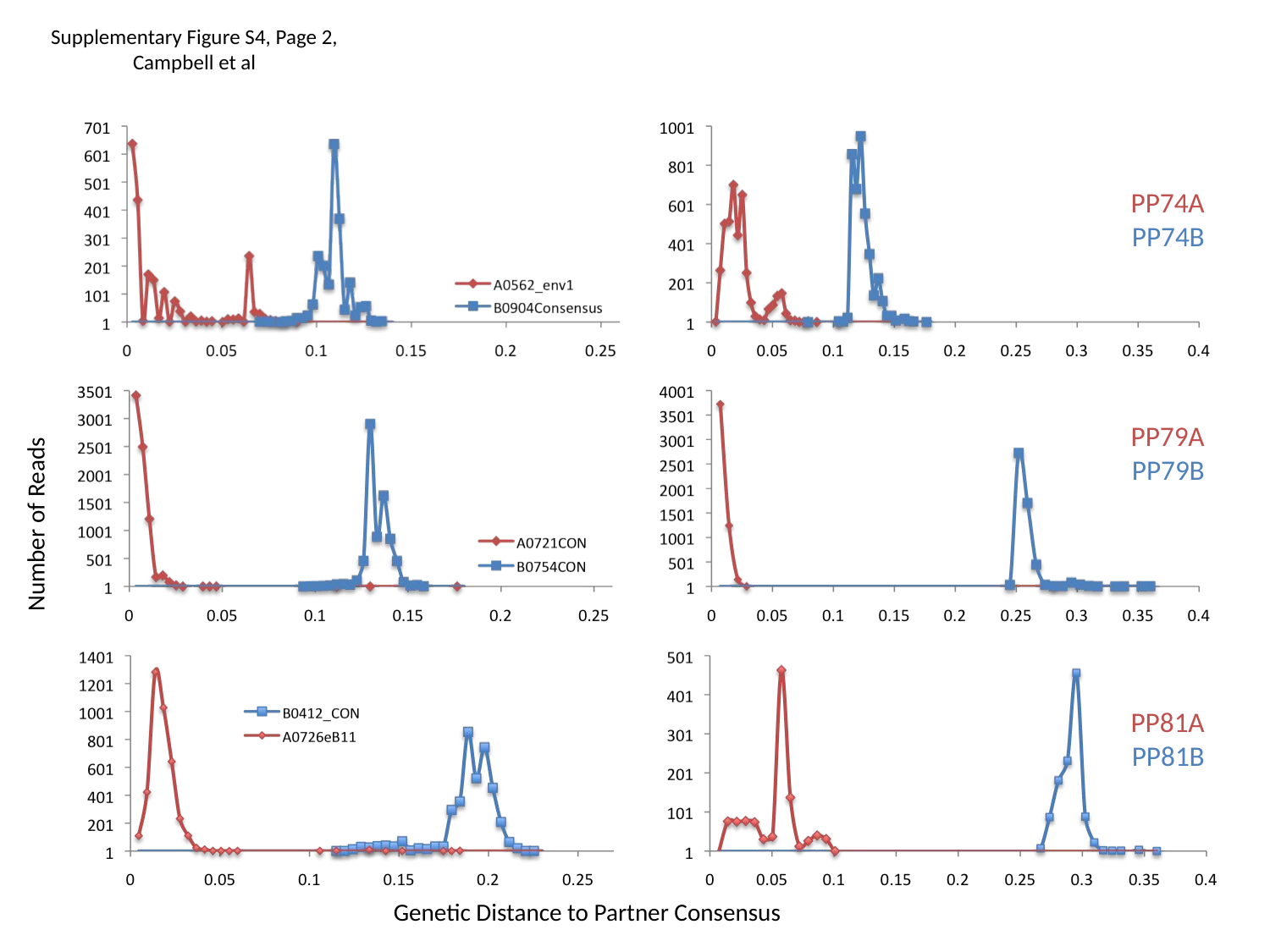

Supplementary Figure S4, Page 2, Campbell et al
PP74A
PP74B
PP79A
PP79B
Number of Reads
PP81A
PP81B
Genetic Distance to Partner Consensus

## Slide 3
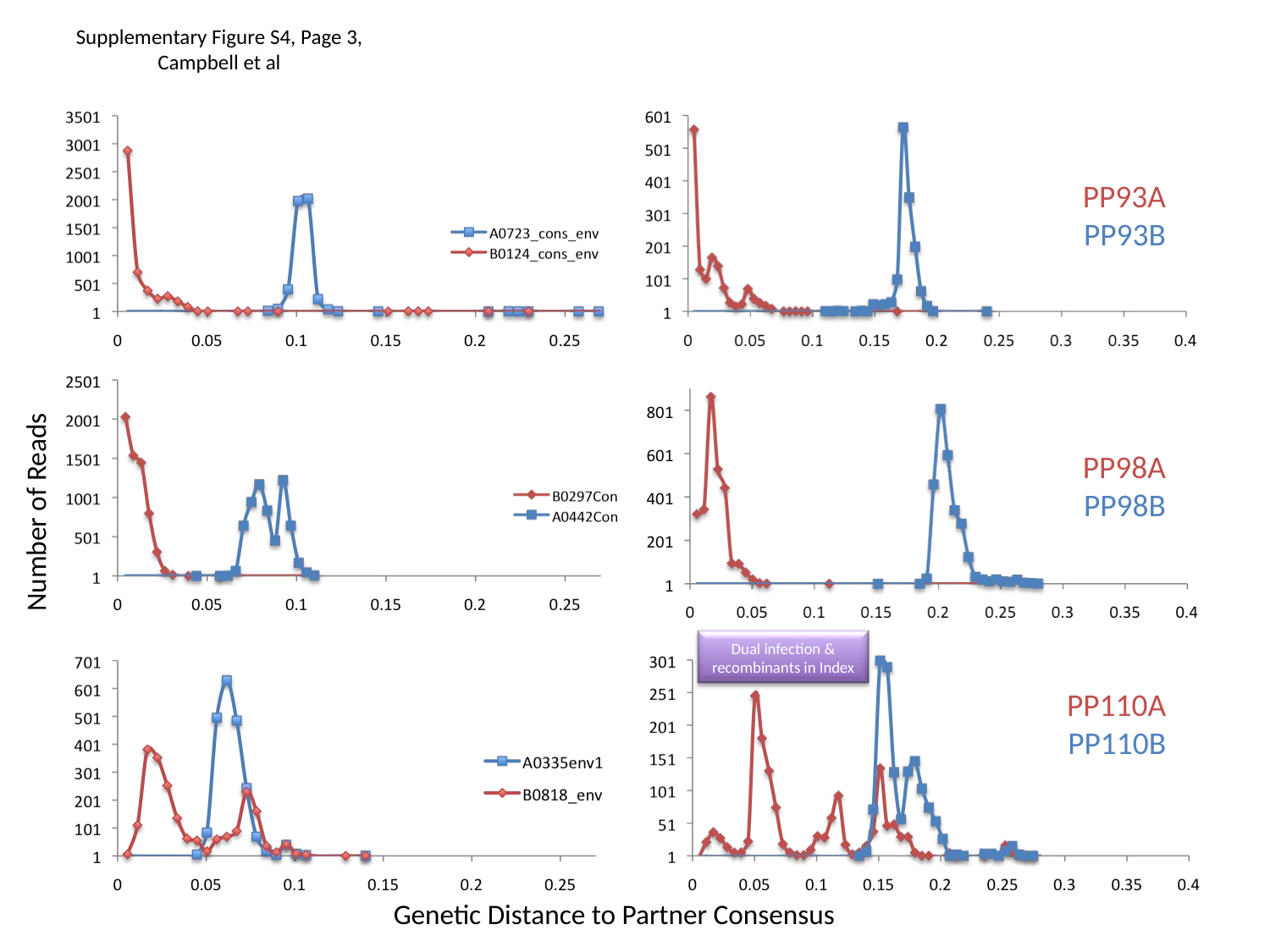

Supplementary Figure S4, Page 3, Campbell et al
PP93A
PP93B
PP98A
PP98B
Number of Reads
Dual infection & recombinants in Index
PP110A
PP110B
Genetic Distance to Partner Consensus

## Slide 4
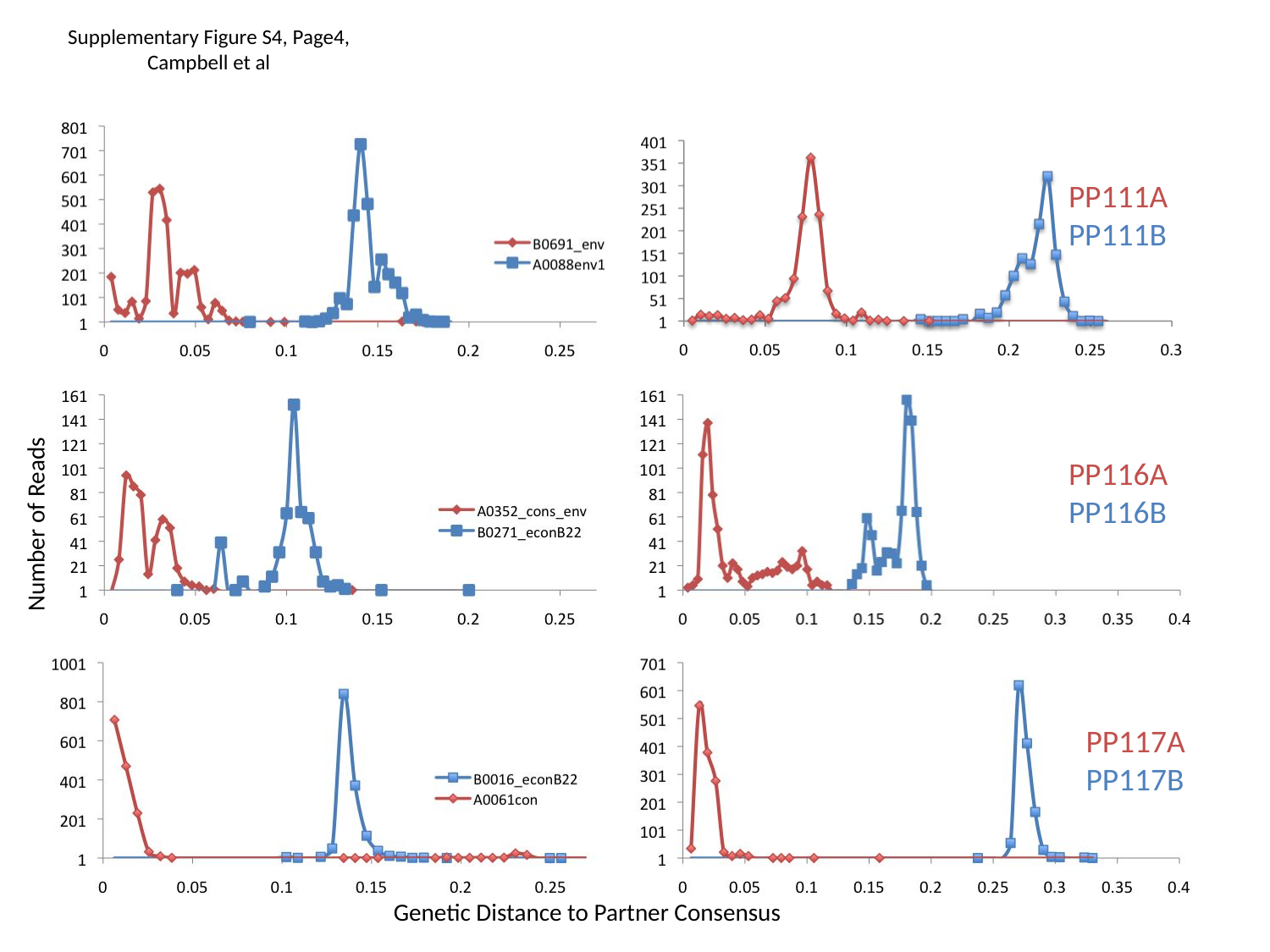

Supplementary Figure S4, Page4, Campbell et al
PP111A
PP111B
PP116A
PP116B
Number of Reads
PP117A
PP117B
Genetic Distance to Partner Consensus
